# Commentary: Anxiety- and Depression-like States Lead to Pronounced Olfactory Deficits and Impaired Adult Neurogenesis in Mice

**DOI:** 10.3389/fnbeh.2016.00130

**Published:** 2016-06-24

**Authors:** Jeremy C. Borniger, Yasmine M. Cissé, Monica M. Gaudier-Diaz, William H. Walker II

**Affiliations:** Neuroscience Graduate Program and Behavioral Neuroendocrinology Group, Department of Neuroscience, The Ohio State University – Wexner Medical CenterColumbus, OH, USA

**Keywords:** glucocorticoids, anxiety, depression, neurogenesis, olfactory bulb

Depression and anxiety disorders are characterized by complex and idiopathic etiologies. One prominent hypothesis, termed “the neurogenesis hypothesis,” posits that stress-induced decreases in hippocampal neurogenesis underlie depressive episodes (Jacobs et al., 2000). Glucocorticoids are primary effectors of the stress response. Acute glucocorticoid exposure enhances cognitive capacity by altering hippocampal synaptic plasticity. In contrast, chronic exposure causes hippocampal atrophy via excessive glutamate, calcium, and oxygen free radical generation. The hippocampus has become central in affective disorder research because blocking hippocampal neurogenesis eliminates some beneficial effects of monoaminergic antidepressants (Surget et al., 2008), whereas increasing neurogenesis reduces glucocorticoid-induced anxiety- and depressive-like responses (Hill et al., 2015).

In contrast, the behavioral consequences of neurogenesis in the olfactory bulb (OB), the other brain structure that integrates new neurons in the adult mammalian brain, remains unspecified. Subventricular zone (SVZ) neuroblasts migrate to the OB along the rostral migratory stream (RMS). Chronic stress affects this migration (e.g., Yang et al., 2011). Olfactory neurogenesis occurs in humans (Curtis et al., 2007); patients with major depression display impaired olfactory function and sensitivity; it is unknown whether these changes are related to glucocorticoid dysregulation and reduced neurogenesis that often accompanies depressive disorders.

A new study Siopi et al. (2016) provides evidence that chronic corticosterone exposure decreases OB cell survival leading to impaired olfactory function. A subset of the observed deficits could be rescued by co-administration of fluoxetine (FLX), a commonly prescribed selective serotonin reuptake inhibitor (SSRI). Specifically, mice administered corticosterone decreased olfactory discrimination, acuity, and memory. FLX in combination with corticosterone restored deficits in olfactory acuity and memory, but not olfactory discrimination. FLX alone impaired these same measures. Together, these data imply that FLX rescues corticosterone-induced deficits, but has a paradoxically detrimental effect when administered in the absence of elevated glucocorticoids. Notably, chronic corticosterone treatment did not impair cell proliferation (Ki67^+^ cells) within the olfactory epithelium or OB sensory afferentation (OMP^+^ cells), suggesting deficits in odor processing rather than detection. Corticosterone administration decreased cell proliferation in the dentate gyrus (DG), but did not affect cell proliferation in the SVZ. These data suggest a different mechanism by which corticosterone affects neuroblasts destined for the OB. To investigate this, survival of newly born neurons maturing in the DG and OB was examined. Glucocorticoids decreased cell survival in both regions; this effect was prevented by co-administration of FLX. Additionally, dendritic complexity of newly integrated neurons increased following FLX administration, potentially contributing to the partially rescued phenotype. Taken together, these results link major depressive disorder and neurogenesis-dependent olfactory deficits within the DG and OB. Understanding the implications of impaired olfactory function and neurogenesis in depressed humans warrants further investigation; additional mechanisms by which glucocorticoids modulate neurogenesis and functional synapse formation should be examined.

The translational significance of these new data is worth considering in the context of vast species differences in rates of olfactory neurogenesis and reliance on olfaction. Rodents rely more heavily on olfaction than humans, and neurogenic rates in the OB are minimal in adult humans (Kam et al., 2009). However, at the anatomical level, there are similarities in neurogenesis across species. Analysis of OBs from patients treated with 5-bromo-2′-deoxyuridine (BrdU) reveal that human progenitor cells from the SVZ also travel through the RMS to the OB where they mature (Curtis et al., 2007). The functional implications of olfactory neurogenesis in depression and anxiety disorders remain understudied; converging evidence suggests that impaired olfactory function predicts disease progression (e.g., Roberts et al., 2016). For example, untreated suicide victims accumulated neural progenitor cells (Doublecortin^+^) in the SVZ and OB relative to psychiatrically healthy people (Maheu et al., 2015). In antidepressant-treated suicide victims, the number of Doublecortin^+^ cells in the SVZ was reduced without simultaneous reductions in the OB. These data suggest that impaired migration and ectopic differentiation leads to neuroblast accumulation in the OB of unmedicated depressed individuals. Antidepressant treatment facilitates neuroblast migration out of the SVZ, but cells that differentiated ectopically prior to treatment persist (Maheu et al., 2015). Contrary to the migration and differentiation impairment hypothesis (Maheu et al., 2015), reduced cell survival underlies rodent deficits in olfactory neurogenesis (Siopi et al., 2016). The differences between clinical and mouse data warrant further studies of depression-induced deficits in olfactory neurogenesis and function.

Although stress-induced increases in glucocorticoids directly regulate neurogenesis, immune cells are also responsible for maintenance of the neurogenic niche (Ziv et al., 2006) and may provide an indirect mechanism through which stress alters neurogenesis. Specifically, microglia are responsible for clearance of apoptotic neuroprogenitors and neuroblasts and are highly responsive to glucocorticoids. Chronic exposure activates microglia and induces a pro-inflammatory phenotype (Nair and Bonneau, 2006) that is ultimately detrimental to neurogenesis (Battista et al., 2006); OB microglia may play a role in regulating neurogenesis in a minimally activated state (Lazarini et al., 2012). Therefore, microglial activation may contribute to the phenotype observed (Siopi et al., 2016). FLX reverses deficits in cell proliferation and survival both in the DG and OB. Serotonin increases cell proliferation and neurogenesis, acting on microglia to reduce phagocytic activity (Krabbe et al., 2012). Thus, decreased cell clearance may account for increased cell survival in mice receiving a combination of corticosterone and FLX (Siopi et al., 2016). Along with increased survival, new neurons in the OB of FLX + corticosterone- and FLX-treated animals increased dendritic length and branching. Synaptic pruning is also regulated by microglia. Examining whether these dendrites are forming functional synapses could provide insight into why FLX-treated animals did not improve in olfactory tests.

In sum, Siopi and colleagues suggest that chronic elevated glucocorticoids reduce hippocampal and olfactory neurogenesis, leading to depressive-like states and impaired olfactory function (Figure 1). Fluoxetine restores hippocampal neurogenesis and cell survival of newly born neurons integrating into the olfactory bulb. We advocate for further studies on the functional significance of human olfactory neurogenesis in depressed patients. Additionally, glial contributions to neurogenesis and synapse formation should be considered in the context of depression and olfactory function.

**Figure 1:**
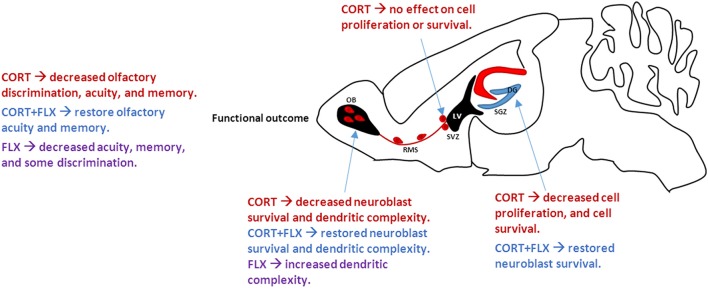
**Effect of CORT and FLX treatment on adult mouse neurogenesis**. CORT treatment decreases cell proliferation and survival in the SGZ, ultimately associated with increased depressive- and anxiety-like behaviors. However, CORT has no effect on SVZ neurogenesis. Neuroblasts travel from the SVZ along the RMS to the OB. CORT treatment decreases neuroblast survival and dendritic complexity in the OB, leading to overall decreased olfactory function. FLX, when co-administered with CORT, restores cell survival and olfactory function. CORT, corticosterone; FLX, fluoxetine; SGZ, subgranular zone; SVZ, subventricular zone; RMS, rostral migratory stream; OB, olfactory bulb; LV, lateral ventricle.

## Author contributions

All authors contributed equally to the writing and review of this manuscript.

## Funding

JB is supported by an OSU Presidential and Pelotonia pre-doctoral fellowship. YC was supported by National Institute of Dental and Craniofacial Research Grant T32DE014320.

### Conflict of interest statement

The authors declare that the research was conducted in the absence of any commercial or financial relationships that could be construed as a potential conflict of interest.
